# Engineered Extracellular Vesicles/Exosomes as a New Tool against Neurodegenerative Diseases

**DOI:** 10.3390/pharmaceutics12060529

**Published:** 2020-06-09

**Authors:** Flavia Ferrantelli, Chiara Chiozzini, Patrizia Leone, Francesco Manfredi, Maurizio Federico

**Affiliations:** National Center for Global Health, Istituto Superiore Di Sanità (ISS), 00161 Rome, Italy; chiara.chiozzini@iss.it (C.C.); patrizia.leone@iss.it (P.L.); francesco.manfredi@iss.it (F.M.)

**Keywords:** neurodegenerative disease therapy, extracellular vesicles/exosomes, intrabodies

## Abstract

Neurodegenerative diseases are commonly generated by intracellular accumulation of misfolded/aggregated mutated proteins. These abnormal protein aggregates impair the functions of mitochondria and induce oxidative stress, thereby resulting in neuronal cell death. In turn, neuronal damage induces chronic inflammation and neurodegeneration. Thus, reducing/eliminating these abnormal protein aggregates is a priority for any anti-neurodegenerative therapeutic approach. Although several antibodies against mutated neuronal proteins have been already developed, how to efficiently deliver them inside the target cells remains an unmet issue. Extracellular vesicles/exosomes incorporating intrabodies against the pathogenic products would be a tool for innovative therapeutic approaches. In this review/perspective article, we identify and describe the major molecular targets associated with neurodegenerative diseases, as well as the antibodies already developed against them. Finally, we propose a novel targeting strategy based on the endogenous engineering of extracellular vesicles/exosomes constitutively released by cells of the central nervous system.

## 1. Introduction

Several neurodegenerative diseases (NDs) arise from cell degeneration induced by the accumulation of misfolded/mutated proteins. Among NDs generated by protein mutations, many disorders are caused by polyglutamine extension, a consequence of the genetic expansion of the CAG trinucleotide repeat. These include Huntington’s disease, spinobulbar muscular atrophy, spinocerebellar ataxia, Machado–Joseph disease, and α-synucleinopathies [1]. Parkinson’s disease (PD), among α-synucleinopathies, is associated with the formation of α-synuclein aggregates referred to as Lewy bodies [2]. In Alzheimer’s disease (AD), both extracellular Amyloid beta (Aβ) plaques and intracellular fibrillary tangles of Tau protein accumulate over time leading to potent neurotoxic effects [3]. Similarly, aggregated/misfolded mutated Cu/Zn superoxide dismutase (SOD1) associate with amyotrophic lateral sclerosis (ALS) [4].

### 1.1. Intrabodies

**Figure 1:**
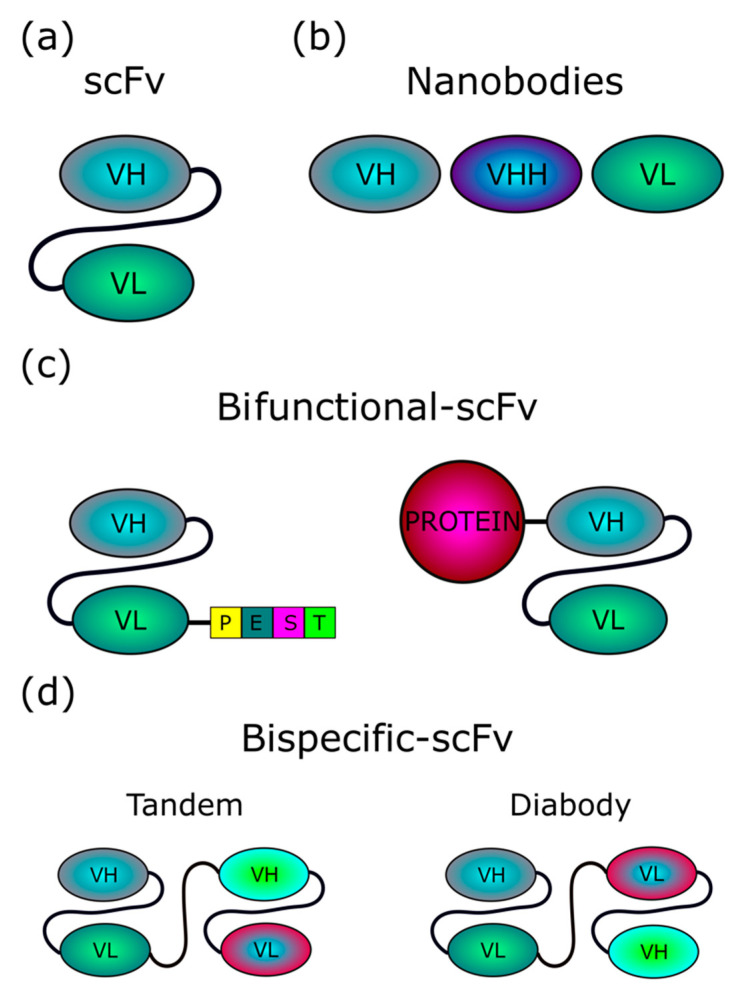
Structure of intrabodies. (**a**) scFvs (~30 kDa) consist of variable regions of heavy (VH) and light (VL) chains of an antibody, joined by a flexible linker, which is usually a 15-aa (Gly_4_Ser)_3_ sequence. Both orientations, VH-linker-VL or VL-linker-VH, are possible. (**b**) Single domain intrabodies (~15 kDa), also termed nanobodies, consist of one single variable domain, and are referred to as VH (or VHH, for nanobodies based on naturally occurring camelid heavy-chain only antibodies), or VL, depending on the variable region included. (**c**) Bifunctional scFvs can be designed by coupling, at either the N- or C-terminus, an scFv with either a protein domain (for instance, PEST degron) able to redirect the scFv-bound target to cellular clearance machinery, or a whole protein, such as the Nef variant (~27 kDa) we propose to exploit in our exosome-based intrabody delivery strategy. (**d**) Bispecific scFvs include two scFvs with different specificity, fused either in tandem or head-to-tail (diabody).

To specifically target such abnormal protein aggregates, a number of antibody fragments that are active intracellularly, called intrabodies, have been recently developed (Table 1). To become intrabodies, in general, antibody portions are specifically modified for localization in intracellular compartments, such as the cytoplasm, the nucleus, mitochondria, or the endoplasmic reticulum (ER) of cells that express them. Intrabodies include single-chain fragment variable antibodies (scFvs). In fact, the antibody Fv region, which is responsible for antibody specificity and binding to a target, can be expressed in the absence of other immunoglobulin domains, and further engineered for the design of therapeutic molecules (Figure 1) [5]. Notably, scFv intrabodies encompass both variable heavy (VH) and light (VL) chains of an antibody, connected by a flexible hinge (Figure 1) [6], and are capable of effectively binding their target antigens. Several other antibody or scFv derivatives against NDs have been developed to be used as intrabodies, such as bifunctional scFvs and bispecific scFvs, as well as camelid VHH, VH, or VL single domain antibodies (nanobodies), which may be more stable, more soluble in the intracellular reducing environment, and have easier access to hidden epitopes than scFvs (Figure 1) [7]. However, despite the proposition of new strategies, the efficient intracellular expression/delivery of these intrabodies remains an unsolved issue.

### 1.2. Extracellular Vesicles

All cells constitutively release lipid bi-layered vesicles of various sizes and with different biogenesis, named extracellular vesicles (EVs). EVs are classified as microvesicles/ectosomes (50–1000 nm) and exosomes (50–200 nm) [47]. Whereas the former shed directly from the plasma membrane, exosomes are nanovesicles that originate intracellularly upon inward invagination of endosome membranes, accumulation of intraluminal vesicles (ILVs) in multivesicular bodies (MVBs), trafficking towards the plasma membrane, and release in the extracellular milieu [48].

Exosomes play many relevant roles in CNS, both in health and in disease. For instance, oligodendrocytes release exosomes loaded with components of the myelin sheaths, likely in response to glutamate secreted by neurons [49]. Also, EVs from microglia stimulate neuron synaptic activity by increasing the sphingolipid metabolism and astrocytes release EVs loaded with synapsin I, i.e., a protein that induces neurite formation and survival in neurons [50]. On the other hand, EVs upload and transmit many CNS pathogenetic products in NDs, like Aβ peptides, α-synuclein, SOD-1, as well as the altered prion protein PrP^SC^ [51,52].

In this review, the genetic/molecular bases of most common NDs are summarized. In addition, an overview of the characterization of scFvs and other intrabodies against functional targets of a number of NDs is provided, as well as current strategies for their intracellular expression/delivery. Finally, the potential and future implementation of a novel approach based on the endogenous engineering of EVs/exosomes that we are developing for the delivery of scFvs into target cells is also described.

## 2. Genetic Basis of Neurodegenerative Diseases

### 2.1. Alzheimer’s Disease

AD is the most widespread cause of dementia [53]. Although the onset of disease mostly occurs after 65 years, an AD subtype (i.e., early-onset AD) can affect younger people [54]. In this case also, molecular signatures of AD are the accumulation of misfolded protein aggregates, which can self-propagate in a prion-like manner, leading to neurotoxicity and cell death [55]. In particular, AD brains accumulate amyloid plaques formed by Aβ(1-42) fibrils generated by the cleavage of the amyloid plaques by both β- and γ-secretases [3,55]. In addition, hyperphosphorylated Tau protein, which normally stabilizes neuronal microtubules, aggregates thereby producing neurofibrillary tangles affecting the intracellular transport machinery of neurons [3]. Both familial and early-onset AD associate with genetic alterations, in particular localized in the amyloid precursor protein, presenilin 1, and presenilin 2 [56].

### 2.2. Parkinson’s Disease

PD is the second most diffuse neurodegenerative pathology after Alzheimer’s [57]. Although the mechanisms underlying the disease pathogenesis remain largely unknown, a number of genetic signatures characterize disease onset. For instance, the A53T mutation in α-synuclein was the first amino acid substitution associated with familial PD to be discovered [58]. Afterward, additional mutations within the α-synuclein N-terminus have been identified. In its physiologic form, this protein forms intracellular fibrils. When mutated, it becomes the major component of Lewy bodies, i.e., macromolecular aggregates having toxic effects on dopaminergic neurons as a consequence of the altered intracellular signaling and defective intracellular trafficking leading, for instance, to autophagy suppression [59,60].

Mutations associated with the death of dopaminergic neurons and PD have been identified in additional six genes, i.e., Parkin, vacuolar protein sorting-35, glucocerebrosidase, PTEN-induced putative kinase, leucine-rich repeat kinase 2, and DJ-1 [61]. Whatever the role possibly played by each mutated product, it is now accepted that either single-or multi-gene mutations are involved in PD pathogenesis, thereby representing potential targets for therapy.

### 2.3. Huntington’s Disease

At least nine human NDs are generated by the expansion of the CAG nucleotide repeats, forming an abnormal polyglutamine extension of neuron proteins [62]. Among these, Huntington’s disease (HD) is an autosomal dominant ND generated by the CAG expansion in the first exon of the Huntingtin gene resulting in a protein with an abnormally long polyglutamine sequence [63]. The N-terminal polyglutamine expansion leads to abnormal protein aggregation and neuron intracellular inclusions ultimately having a cytotoxic effect on neurons [64].

### 2.4. Amyotrophic Lateral Sclerosis

ALS is a fatal neurodegenerative disorder characterized by the progressive loss of motor neurons, ultimately leading to paralysis and death [65]. To date, nearly 200 mutations have been described to be associated with familial ALS [66]. Similarly to other NDs, superoxide dismutase 1 (SOD1) toxic effects are at least in part due to the generation of intracellular aggregates of misfolded protein. The most common mutations found in the *SOD1* gene are D90A, A4V, and G93A. More recently, mutations in more than 20 genes have been linked to both familial and sporadic ALS, including *C9orf72*, *TARDBP* (*TDP43*), and *FUS* [67].

### 2.5. Prion Disorders

A misfolded form of the prion protein (PrP) is at the basis of a number of human NDs, including Creutzfeldt–Jakob disease, Kuru, as well as spongiform encephalitis and scrapie in bovines and sheep, respectively. Native PrP (PrP^C^) is expressed in all tissues, but particularly in the CNS [68]. Misfolded PrP^C^ (PrP^Sc^) is infectious and able to catalyze the conversion of PrP^C^ into PrP^Sc^, and is, therefore, responsible for both transmission and pathogenesis of the disease [55,69,70]. PrP^Sc^ aggregates generate amyloid fibers that heavily affect nervous functions [55].

## 3. Intrabodies against ND Targets

Intrabodies can be specifically designed, for instance, to accumulate in the cell where they are expressed, by not including a secretory signal. Alternatively, they can be engineered to be either released extracellularly for bystander cells uptake or uploaded as cargoes into extracellular vesicles, depending on the strategy to be developed for their therapeutic application.

The rationale behind the use of antibodies and, more specifically, intrabodies as an approach against NDs becomes clear when considering the particular pathogenesis, with intracellularly accumulated misfolded proteins, which might be targeted in order to accelerate their turnover, block post-translational pathogenic modifications or cleavage, and/or modify their subcellular compartmentalization.

### 3.1. Antibody Targets for Alzheimer’s Disease

Phase III clinical trials with at least four anti-Aβ antibodies failed to improve AD symptoms possibly because of the advanced disease stage of the treated patients [71,72]. Nonetheless, in the future, with early treatment, both early-diagnosed and genetic AD cases might benefit from this approach. In fact, passive immunotherapy with nanobodies showed some promise in preclinical studies, as their small size may permit them to access brain tissue more effectively than conventional antibody-based therapies. Moreover, the nanobody technology may allow the combination of multiple functions into the same molecule and nanobodies are considered safer than full-size antibodies, which may cause a number of serious adverse effects [73].

To date, a nanobody specific to Aβ fibrils selected from a phage display a fully synthetic library of camelid VHH (heavy chain variable region)-domains [74] and other anti-Aβ intrabodies have been described [8,9]. In particular, an AAV-delivered scFv intrabody was reported to decrease AD pathology in a mouse model for AD, both at the molecular and cognitive levels [9].

Anti-Aβ scFv can prevent fibril aggregation in a cell-free system, and toxicity in neuronal cells [75,76]. Furthermore, scFv intrabodies specific for a cleavage site in the Aβ precursor protein prevented the generation of Aβ [77]. These observations indicate that Aβ is produced intracellularly, before accumulating outside the cell, thus the formation process and intra- and extracellular accumulation may be effectively targeted by intrabodies. The binding of scFvs or nanobodies to extracellular Aβ may also be beneficial to prevent deposition, plaque formation, and pathogenesis, although, as such, these would not be truly considered intrabodies [78].

Interestingly, a bispecific scFv construct both blocking the cleavage site of β-secretase and increasing α-secretase cleavage showed promise in a mouse model [79]. Such scFv was delivered through AAV gene therapy to the liver and, being engineered to cross the blood–brain barrier (BBB), exerted its function upon entering target cells from the outside. Long-term studies will be necessary to determine whether a similar bispecific intrabody-based approach can overcome the issue of irreversible changes that may occur during the intrabody-antigen dissociation phase, as also reported for other NDs (see below).

### 3.2. Antibody Targets for Parkinson’s Disease

Misfolded α-synuclein accumulates inside and outside the cell and causes PD progression. For this reason, despite the small size and the partially unfolded structure, both intracellular and extracellular α-synuclein monomers have been considered as therapeutic targets, in particular the non-amyloid component (NAC) hydrophobic interaction region, which is critical for misfolding and pathogenic aggregation of α-synuclein [17,80,81]. Efforts to generate antibodies specific to the NAC region in vivo were unsuccessful, likely because this domain is not exposed within the folded protein. Therefore, the search for therapeutic molecules shifted to the screening of libraries of antibody fragments with peptides derived from α-synuclein NAC. Intrabodies initially selected from a yeast surface-display library of human scFvs were quite disappointing, as those identified as the best binders in vitro, when tested in a cell-based assay, resulted not protective, thus proving dysfunctional. This was due to modifications undergone in the intracellular reducing environment, possibly leading to insolubility, and/or to other reasons related to proteostatic stress in cells affected by the disease. The lack of availability of cognate epitope in the context of the protein target in vivo would also account for the observed unsatisfactory results. In some instances, intrabody modifications to obtain a bispecific molecule, such as a proteasome targeting signal, augmented overall protein solubility thanks to an increase in negative charge, thus improving intrabody performance in vivo [10,16].

Full-length α-synuclein protein was also used in an immunization and phage-display library production/selection approach that resulted in the identification of a pool of VHH nanobodies. Among these, Nbsyn2 and Nbsyn87 were characterized and found specific for an α-synuclein C-terminus [19]. This domain of α-synuclein can undergo post-translational modifications that are involved in pathogenic protein misfolding [82], therefore, it is a potential therapeutic target. Yet, only Nbsyn87 proved able to interfere with mutant α-synuclein aggregation in vitro, whereas Nbyn2, specific for a more C-terminal and longer peptide epitope, did not [83].

When both Nbsyn87 camelid VHH and VH14 human VH nanobodies were fused to the C-terminal of mouse ornithine decarboxylase (mODC) PEST degron (a proline, aspartate or glutamate, serine, and threonine motif that modulates protein degradation), α-synuclein aggregation was decreased, its clearance was augmented, and α-synuclein overexpression-induced toxicity in ST14A cells was reduced. Nbsyn87 showed the strongest α-synuclein reduction, whereas VH14 conferred the best survival benefits to the cells [16]. In an in vivo model of viral gene therapy of α-synuclein overexpression in rats [20], both PEST-fused Nbsyn87 and VH14 nanobodies showed protective effects against pathological α-synuclein aggregation, mostly in terms of a reduction in phosphorylated Serine-129 α-synuclein. VH14-PEST nanobody was somewhat more protective than Nbsyn87-PEST, possibly because the latter induced localized inflammation that may have affected overall protection. Initial studies have also been conducted with other scFvs and nanobodies, which were tested against oligomeric, protofibrillar, and fibrillar forms of α-synuclein in vitro [84,85].

### 3.3. Antibody Targets for Huntington’s Disease

The approach based on intrabodies is reasonably feasible also in HD, since abnormally folded polyQ protein fragments accumulate inside the cell. Thus, it is conceivable to target and prevent the early stages of the disease by altering the initial misfolding of the expanded polyQ tract and its subcellular compartmentalization. In particular, it would be of therapeutic relevance to contrast its accumulation in cell nuclei and interaction with other abnormal proteins, as well as promoting its turnover [86,87]. Based on this rationale, specific anti-HTT intrabodies (scFvs and single-domain antibodies) were initially selected from phage or yeast surface-display libraries, as well as from hybridoma cell lines to target the N-terminal fragment of the mutant HTT (mHTT) protein, presenting an extended, misfolded polyQ. Unfortunately, these anti-fibrillar intrabodies stabilized and enlarged the size of pathogenic fibrils, and this approach turned out to increase their toxicity [18,36]. An alternative strategy has been therefore explored, aimed at targeting both the N- and the prolin-rich C-terminal domains flanking the polyQ, in order to alter its context and neutralize mHTT toxicity.

In line with this, scFvC4 was the first anti-HTT intrabody selected from sub-pools of a naïve human spleen phage-display library against the unmodified HTT N-terminal 1–17 peptide [25]. It was proven able to preferentially disaggregate pathogenic mHTT Exon1 fibrils [27] rather than the full-length wild type HTT, thus leaving the latter able to exert its normal functions in vivo [88].

Selected candidate intrabodies were tested as transgenes in fly and mouse models with some promising results [26,30,89], yet the effect of treatment with scFvC4 diminished over time because of the formation of large insoluble complexes, which escape mHTT Exon1 clearance by intrabodies. This effect was likely due to PolyQ becoming rapidly fibrillar during the brief periods of dissociation of the intrabody. Therefore, newer approaches aim at developing bifunctional intrabodies, which facilitate the turnover of targeted proteins before complex dissociation occurs, by exploiting cellular clearance mechanisms to rapidly target bound intrabody–antigen complexes [21,90,91]. In such intrabodies, for instance, the scFvC4 C-terminus is coupled with the mODC PEST degron to redirect intrabody–antigen complexes to the proteasome [21]. Alternatively, bifunctional anti-Huntingtin intrabodies have been developed by fusing heat shock protein 70 (HSC70) protein binding motifs (KFERQ and VKKDQ) to polyQ binding protein (QBP1), derived from a monoclonal antibody specific to expanded polyglutamine repeats [92], to direct mHTT to the chaperone-mediated autophagy (CMA) lysosomal degradation pathway [90,91]. Further studies will be necessary to optimize the design of bifunctional antibodies taking into account general stability, delivery strategies, and safety.

Another anti-N-terminus intrabody was selected against HTT AA1-20 from the non-immune human yeast surface-display library and engineered as a single domain intrabody (VL)12.3, which had shown full binding activity [31]. In vivo studies in mouse models [33] showed controversial results, with VL12.3 administration either improving animal behavior and decreasing neuropathology or actually inducing a slight acceleration of the disease. The latter effect was likely due to high antigen–antibody complexes in nuclei of transduced cells [33].

Several monoclonal antibodies and intrabodies specific to the PolyQ C-terminal region, which encompasses a polyproline (PolyP)-rich region involved in the modulation of PolyQ aggregation were also investigated [93,94]. In particular, an scFv derived from PolyP-specific MW7 monoclonal antibody proved able to reduce mHTT aggregation and to enhance cell survival in an in vitro HD model [36]. In addition, anti-proline-rich region single-domain VL intrabodies Happ1 and Happ3 obtained from a non-immune human recombinant scFV phage library [33] were able to prevent aggregation and toxicity at lower doses than scFvMW7 [33]. All three intrabodies were capable of increasing mHTT Exon1 clearance in HEK293 cells, possibly by keeping their bound target soluble and available for regular turnover processes.

An additional anti-PolyQ C-terminus intrabody, INT41, showed some promise in short-term studies in both in vitro and in vivo HD models, in terms of phenotype amelioration, decrease of protein aggregation, and cognition improvement. Longer-term experiments will be required to assess whether such improvements last over time. Promising results were also obtained with another monoclonal antibody-derived scFv (SCFV48), binding a more C-terminal epitope, which counteracted the cytoplasmic, yet not the nuclear, toxicity of the mHTT Exon 1 in HEK293 cells [38].

### 3.4. Antibody Targets for Amyotrophic Lateral Sclerosis

ALS candidate intrabody targets SOD1, C9orf72, and TDP43 are being evaluated in preclinical studies. In particular, phage-display library-selected anti-SOD1 scFvs, administered intravenously in the G93A mutant SOD1 mouse model of ALS, displayed expression in motor neurons and astrocytes and had beneficial effects [40,41]. A monoclonal antibody-derived scFv (D3H5), specific for misfolded SOD1, administered by intrathecal injection, also induced delayed disease onset and prolonged survival in G93A mutant SOD1 mice in a dose-response manner, proportional to D3H5 scFv levels in spinal cord motor neurons [42].

Another monoclonal antibody-derived scFv, specific for the TDP-43 RNA recognition motif 1 (RRM1) domain, proved protective against TDP-43 proteinopathy both in cell cultures and upon intrathecal/intracranial delivery [95]. Despite the absence of fused degron sequences, a slight increase in target protein turnover was also reported. Both intracellular and secreted scFv taken-up by cells from the outside are likely to contribute to counteracting pathological inflammation.

### 3.5. Targets for Prion Disorders

Although PrP misfolding is not correlated with known genetic mutations, the different conformations of native compared to pathogenic altered protein allowed the isolation of antibodies specifically recognizing PrP^Sc^. In particular, 8H4- and 8F9-scFvs, directed against different PrP epitopes, were targeted to the secretory compartment of mammalian cells by ER retention signal KDEL-tagging. In transmissible spongiform encephalopathy (TSE), these scFvs were able to prevent the conversion of normal PrP^C^ to pathological isoforms and avoid the accumulation of the misfolded protein, therefore diminishing its infectivity [43,44]. In vivo studies also showed that anti-PrP KDEL-fused intrabodies can prevent scrapie infectivity in mice [44]. On the other hand, crystallographic studies based on anti-PrP scFvs and nanobodies also helped characterize the bovine PrP unstructured domain [45,46,96].

## 4. Current Strategies for Intrabody Delivery into Cells

### 4.1. Extracellular Vesicles/Exosomes as a Tool for scFv Delivery

EV manipulation is now considered a rather promising approach for the delivery of drugs and biologically active macromolecules. Stability, biocompatibility, and capacity to cross the BBB are significant advantages of EV-based delivery strategies. Consistently, the use of EVs has been successful for the delivery of small molecules, micro-, non-coding, and messenger RNAs, and proteins [97].

**Figure 2:**
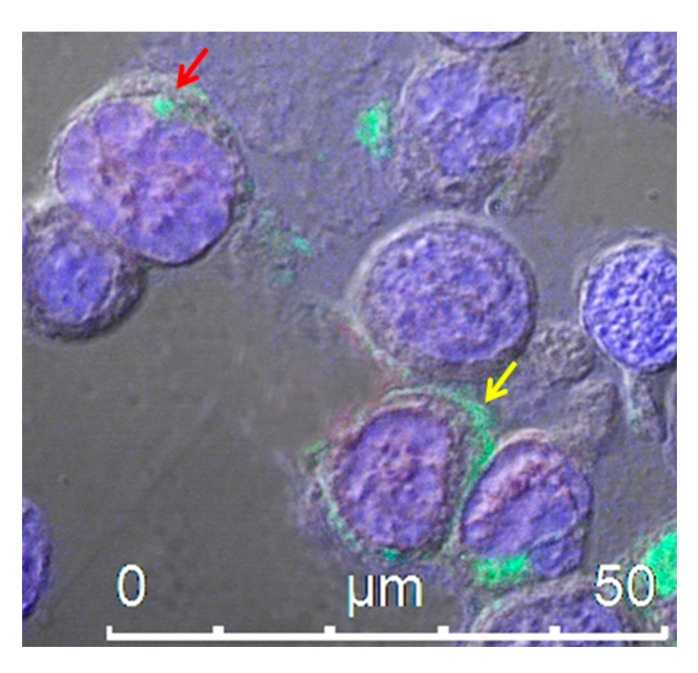
Confocal microscope analysis of HEK293T cells transfected with an expression vector encoding Nef^mut^ fused to an scFv, labeled with an anti-Nef monoclonal antibody and Alexa 488-conjugated anti-mouse IgG antibody. The picture shows intracellular localization of the fusion protein product in intracytoplasmic vesicle aggregates (red arrow) and at the plasma membrane (yellow arrow). DAPI (blue fluorescence) was used to highlight cell nuclei. Scale bar is shown.

**Figure 3:**
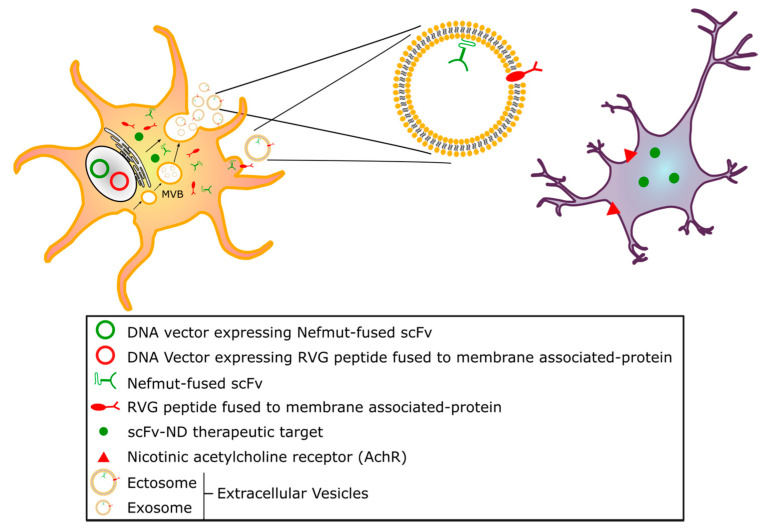
Exosome model for delivering intrabodies against NDs. The model we propose relies on Nef^mut^-based endogenously engineered extracellular vesicles (EVs), produced in vivo by cells expressing vectors encoding a Nef^mut^-scFv fusion intrabody specific for the ND molecular target of interest. Such a bifunctional intrabody may bind its intracellular target both in the DNA-expressing cells and in bystander cells, upon release and cell-to-cell transfer of Nef^mut^-scFv-engineered EVs, by exploiting Nef^mut^ ability to be uploaded into exosomes in large amounts. To enhance the targeting of cells that are relevant to ND pathogenesis, EVs could be doubly engineered with an additional fusion product encompassing a membrane-associated protein and a protein domain specifically able to bind CNS cellular targets, such as, for instance, the RVG-29 peptide, which binds nicotinic AchR.

Proteins can be uploaded by EVs either as constituents of the limiting membrane or incorporated into the lumen, as described for human immunodeficiency virus (HIV)-1 Nef protein [98]. We identified a Nef mutant (referred to as Nef^mut^), which is incorporated into EVs/exosomes at very high levels even when fused with foreign proteins, meanwhile lacking all the biological activities of the wild-type counterpart [99,100]. These unique features allowed us to envision and obtain the proof-of-concept for a novel strategy for intracellular scFv delivery, based on EVs engineered by Nef^mut^ fused at its C-terminus with an scFv. In this conformation, the scFv incorporated into EVs remains protected from modifier/degradation factors present in the extracellular milieu. In the cell, the Nef^mut^/scFv fusion product mostly localizes at the plasma membrane and in intracytoplasmic vesicle aggregates (Figure 2). Using an scFv binding the oncogenic HPV16-E7 fused with Nef^mut^, we demonstrated that the scFv moiety is functional upon delivery in target cells [101]. Hence, engineering EVs with scFvs has the potential to be exploited as a novel therapeutic approach to target intracellular pathogenic factors in NDs. Nonetheless, in view of a translation into the clinic, severe technical hindrances still need to be solved, including the effective delivery of sufficient amounts of engineered exosomes at CNS, industrial manufacturing, cost of production, and storage. To overcome most part of these hurdles, we propose a strategy through which the engineered exosomes are produced by CNS cells of the patient (Figure 3).

### 4.2. Endogenously Engineered Exosomes: Potential and Further Development

As an alternative to the in vitro production of Nef^mut^-based engineered EVs, we developed an in vivo method to engineer the EVs constitutively released by muscle cells [102]. This strategy relies on the intracellular expression of a DNA vector expressing the product of the fusion between Nef^mut^ and the gene of interest. By entering target cells, these engineered EVs allow the intracellular delivery of their cargo. When Nef^mut^ is fused with sequences coding for an scFv, intracellular antigens can be targeted both in DNA vector expressing cells and in bystanders, through cell-to-cell transfer of scFv-engineered EVs (Figure 3).

To attain the highest concentration of anti-ND therapeutic exosomes in the relevant tissue (CNS), at least two conditions should be met: i) engineered exosomes not encountering physiological barriers, which may limit their diffusion, except the plasma membrane of target cells, and ii) therapeutic exosomes preferably entering cell types relevant to disease pathogenesis. As they can spontaneously cross the BBB, exosome surface engineering with the RVG peptide, i.e., a 29-amino acid peptide derived from the neurotropic rabies virus glycoprotein binding the nicotinic acetylcholine receptor (AchR), could increase their delivery to CNS [103,104,105] and may be exploited when adapting our intrabody delivery strategy to ND treatment.

### 4.3. Delivery using Gene Therapy

Antibody fragments can be delivered as genes in the CNS thanks to advancements made in the past few years in the field of gene therapy for the nervous system, which include clinical trials for gene replacement and FDA-approved gene therapy for spinal muscular atrophy [106]. Although the expression of genes of interest in the CNS has been achieved by several methods, the use of viral vectors still appears to be the best way for in vivo transduction of nervous cells. Vectors derived from adenoviruses, herpesviruses, and lentiviruses have proven useful to transduce CNS cells. However, because of their safety profile and genetic simplicity, vectors derived from adeno-associated viruses (AAVs) can be considered the best option for the delivery of genes of interest in CNS [107,108]. AAVs are small, non-enveloped virions of about 20 nm in diameter, with an icosahedral protein capsid containing an approximately 4.8 Kb linear single-stranded DNA. AVVs belong to the Parvoviridae family and depend on the coinfection of helper viruses (adenovirus, herpesvirus) for replication in host cells. In their absence, AAVs may stably integrate into the host cell, albeit at a relatively low frequency, and remain quiescent. Both wild-type AAVs and recombinant AAVs (rAAVs) used for gene therapy do not cause any known associated pathologies and cause a very mild immune response [109].

AAV-delivered transgene expression is essentially permanent in non-dividing cells. In fact, several studies showed that they are expressed for more than six months in the mouse brain [110] and can persist in other tissues for at least six years in primates [111]. Importantly, a gene therapy trial has shown that the therapeutic effects of AAV-delivered transgenes can persist for at least 10 years in the human brain [112].

Recombinant AAVs can be engineered to target different cell types and tissues. Several variant capsids have been described with potential for delivering intrabodies in neurodegenerative diseases [113,114,115], with AAV9 being the best characterized for intrathecal administration. By gene therapy, intrabodies can be delivered directly to the brain or be administered in the periphery with a viral vector capable of entering the brain to target specific cells. For all these characteristics, AAVs are also a good candidate for delivery in the CNS of the product of the fusion between Nef^mut^ and an anti-ND scFv.

The therapeutic effect of engineered EVs emerging from transduced neuronal cells would also benefit from precise cell targeting. This might be easily achieved by co-expressing with the Nef^mut^-scFv fusion protein, another chimeric product including portions of proteins that strongly associate with exosome membranes, such as the transmembrane domain of platelet-derived growth factor receptor (PDGF-R) or lactadherin C1-C2 domains, and ligands that specifically bind the cell type of interest. For instance, with glycoprotein CD24 being a potential marker of cell populations affected in PD [116], the engineering of exosomes with both a membrane protein fused to a humanized anti-CD24 scFv and Nef^mut^-anti-ND scFv would ensure precise targeting of the therapeutic scFv to CD24-expressing cells.

With these premises, we feel that the combination of gene-therapy and EV-based nanotechnologies may offer an unprecedented opportunity to counteract still untreatable NDs.

## 5. Conclusions

EVs/exosomes are considered a smart tool for drug delivery. Stability, biocompatibility, stealth ability in biologic fluids, as well as the documented capacity to cross the BBB are significant advantages of EV-based delivery systems. However, at present, the use of exosomes in therapy is difficult because of a number of yet unresolved issues, such as the efficiency of exosome engineering, cost-effectiveness, safety, and biodistribution/pharmacokinetics. The strategy we developed would overcome at least part of these difficulties. Furthermore, engineering EVs with ligands of specific cell receptors is feasible and would represent a critical step forward towards a precise intervention against ND mediators.

Apart from our proposal, the rapid evolution towards even more sophisticated and reliable EV-based nanotechnologies combined with the antibody-based technologies holds much promise to fight still untreatable diseases, included NDs, tumors, and infectious diseases.

## Figures and Tables

**Table 1:** Intrabodies against neurodegenerative diseases.

**Alzheimer’s Disease Target: β-amyloid Protein**				
**Antigen**	**Species**	**Intrabody**	**Experimental Phase**	**Reference(s)**
Aβ1–42	scFv	scFvAβ^IB^	Preclinical in mice	[8]
Aβ	scFv	Aβ-scFv	Preclinical in mice	[9]
**Parkinson’s Disease Target: α-synuclein Protein**				
**Antigen**	**Species**	**Intrabody**	**Experimental Phase**	**Reference(s)**
Oligomeric α-synuclein	Human scFv	D5	Preclinical in mice	[10,11,12,13]
Oligomeric α-synuclein	Human scFv	10H	Preclinical in mice	[10,12,14]
Pan-specific α-synuclein	Human scFv	D10	Preclinical in mice	[10,12,15]
Hydrophobic non-amyloid component (NAC) of α-synuclein, AA53–95	Human nanobody (VH)	VH14 (NAC14)	Preclinical in rats	[10,16,17]
α-synuclein AA53–87	Human scFv	NAC32	In vitro in cell culture	[17]
Fibrillar α-synuclein	Human scFv	6E	In vitro in cell culture	[11,18]
α-synuclein, AA118–131	Camelid nanobody (VHH)	Nbsyn2	In vitro in cell culture	[16,19]
α-synuclein, AA137-140	Camelid nanobody (VHH)	Nbsyn87	Preclinical in rats	[16,19,20]
**Huntington’s Disease Target: Huntingtin (HTT) Protein**				
**Antigen**	**Species**	**Intrabody**	**Experimental Phase**	**Reference(s)**
HTT-N17 (PolyQ N-term AA1-17)	Human scFv	scFvC4	Preclinical in mice	[11,21,22,23,24,25,26,27,28,29,30]
HTT-N20 (PolyQ N-term, AA1-20)	Human VL	VL12.3	Preclinical in mice	[22,31,32,33,34,35]
HTT-polyQ	Mouse scFv	MW1	In vitro in cell culture	[36,37]
HTT-polyQ	Mouse scFv	MW2	In vitro in cell culture	[36,37]
HTT-PRR	Mouse scFv	MW7	Ex vivo in brain tissue	[33,36,37]
HTT-PRR	Human VL	Happ1	Preclinical in mice	[33,34]
HTT-PRR	Human VL	Happ3	Ex vivo in brain tissue	[33]
HTT-Exon1	Mouse scFv	EM48	Preclinical in mice	[38]
HTT-PRR	Human scFv	INT41	Preclinical in mice	[39]
Fibrillar mHTT	Human scFv	6E	In vitro in cell culture	[18,21]
**Amyotrophic Lateral Sclerosis (ALS) Disease Target: SOD1 Protein**				
**Antigen**	**Species**	**Intrabody**	**Experimental Phase**	**Reference(s)**
SOD1	Human scFv	B1	Preclinical in mice	[40,41]
SOD1	Human scFv	B12	Preclinical in mice	[40,41]
G93A human SOD1	Mouse scFv	D3H5	Preclinical in mice	[42]
**Prion Disorder Target: Prion Protein (PrP)**				
**Antigen**	**Species**	**Intrabody**	**Experimental Phase**	**Reference(s)**
Cellular PrP	Mouse scFv	8H4	Preclinical in mice	[43,44]
Cellular PrP	Mouse scFv	8F9	In vitro in cell culture	[43]
Cellular PrP	Camelid nanobody (VHH)	Nb_PrP_01	In vitro in crystallography studies	[45,46]
Cellular PrP AA123–125, 164–170, and 174–185	Camelid nanobody (VHH)	Nb484	In vitro in crystallography studies	[46]
